# 

**Published:** 1885-12

**Authors:** 


### CONTENTS:

Art. I—Some of the Physiological and Theraputical Properties of Alcohol, By J. P. Stevens, M. D. .... 337
Art. II—Excision v. Extraction, By Mr. C. Spence Bate, F. R S. .... 348
Art. III—Pulpless Teeth, By Chas F. Ives, M. D. S. .... 354
Art. IV—The Amalgam Question, By J. Hardman. .... 360
Art. V—Discoloration of Gold Fillings, By S. B. Palmer .... 366
Art. VI—Treatment of Deep Seated Abscesses without External Incision By John S. Marshall .... 370
Maryland State Dental Association .... 374

### EDITORIAL, ETC.

The Section of Oral and Dental Surgery in the International Medical Congress .... 375

Correction .... 382

### OBITUARY.

Dr. John P. Holmes .... 383
Dr. John M. Riggs .... 383

### MONTHLY SUMMARY.

Sore Mouth Under Plates .... 384

